# 2D Ruddlesden–Popper Perovskites with Polymer Additive as Stable and Transparent Optoelectronic Materials for Building-Integrated Applications

**DOI:** 10.3390/nano14141184

**Published:** 2024-07-11

**Authors:** Adianne Alamban, Muneeza Ahmad, Nicholas Rolston

**Affiliations:** Renewable Energy Materials and Devices Lab, School of Electrical, Computer and Energy Engineering (ECEE), Arizona State University, Tempe, AZ 85284, USA; aalamban@asu.edu (A.A.); mahmad15@asu.edu (M.A.)

**Keywords:** perovskite, 2-dimensional perovskite, structure, strain, semitransparent, wide bandgap

## Abstract

We report on the use of 2D Ruddlesden–Popper (RP) perovskites as optoelectronic materials in building-integrated applications, addressing the challenge of balancing transparency, photoluminescence, and stability. With the addition of polyvinylpyrrolidone (PVP), the 2D RP films exhibit superior transparency compared to their 3D counterparts with an average visible transmittance (AVT) greater than 50% and photoluminescence stability under continuous illumination and 85 °C heat for up to 100 h as bare, unencapsulated films. Structural investigations show a stress relaxation in the 3D perovskite films after degradation from thermal aging that is not observed in the 2D RP films, which retain their phase after thermal and light aging. We also demonstrate ultrasmooth, wide-bandgap 2D Dion–Jacobson (DJ) films with PVP incorporation up to 2.95 eV, an AVT above 70%, and roughnesses of ~2 nm. These findings contribute to the development of next-generation solar materials, paving the way for their integration into built structures.

## 1. Introduction

Organic-inorganic metal halide perovskites are a rising class of semiconductor materials that have garnered the interest of the scientific community for next-generation optoelectronic technologies ranging from light-emitting diodes [1] to photo- and X-ray detectors [2,3] to lasers [4] to memory devices [5]. These offer numerous opportunities in terms of possible applications of the material system; however, one aspect is particularly well-suited to perovskites based on their compatibility with building-integrated photovoltaics (BIPV). BIPV is a subclass of photovoltaic (PV) systems that integrate into built structures such as facades, roofs, or windows [6]. Such use cases enable local energy generation, which can alleviate electricity distribution costs. In particular, solar windows are a facet of BIPV that leverages the properties of semitransparent PV devices to function simultaneously as glazing and as energy generators. Their benefit is most apparent in urban environments, where the façade–roof ratio is substantially higher [7]. Incumbent technologies based on Si or CdTe dominate the market, but their optical properties are difficult to tune [8]. To usher in the widespread adoption of BIPV windows, both transparency and stability must be achieved.

Perovskite-based BIPVs prove promising in this regard, having achieved strong light utilization efficiencies (LUE = Power Conversion Efficiency (PCE) × Average Visible Transmittance (AVT))—surpassing 5.7% [9]. In addition to highly reported PCE values over 25% [10], perovskites offer ease and rapidity of fabrication, low-cost starting materials, and easier tuning of transparency. Thus, perovskites have emerged as ideal contenders for developing stable and transparent absorbers for BIPV applications [11].

A critical aspect of developing window-based BIPVs is controlling the transparency of the films. Bandgap engineering provides an avenue in which this can easily be achieved. The tunable components of the ABX_3_ structure of perovskites enable engineering of the bandgap over a wide range from ~1.2 eV to >3 eV based on the components used [12]. Achieving higher-bandgap perovskite films is commonly done through partial X-site substitution of iodine with a smaller bromine anion with a smaller ionic radius [13]. At higher bandgaps of ~2 eV, there is a significant increase in transparency while maintaining a theoretical Shockley–Queisser power conversion efficiency of >20% [14]. However, mixed-halide iodide–bromide perovskites routinely face halide segregation under light exposure and susceptibility to moisture and heat-based decomposition [15,16].

The perovskite material’s propensity to degrade is a significant barrier to its widespread adoption as a PV technology. This is particularly true for BIPV applications, as building-integrated solar modules must have comparable lifetimes with window glass—over 20 years—to be commercially viable. To that end, a large and growing body of research demonstrates that two-dimensional (2D) perovskites exhibit enhanced stability compared to 3D perovskites [17]. This is partly due to mechanisms such as surface passivation, moisture resistance, and ion migration suppression characteristics brought on by the presence of bulky spacer cations in the perovskite structure [18].

**Figure 1:**
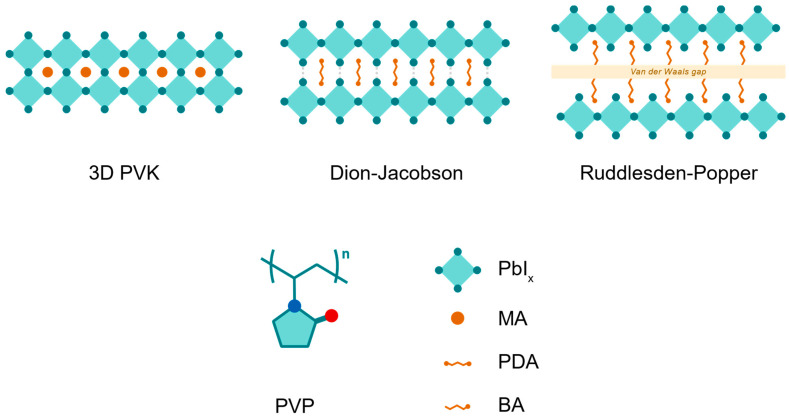
Schematic of the structure of 3D perovskites, n = 1 Dion-Jacobson (DJ) and n = 1 Ruddlesden-Popper (RP) perovskites, and the polymer additive polyvinylpyrrolidone (PVP), respectively. The gray dashed lines in the DJ schematic represent hydrogen bonding between slabs of lead iodide octahedra. The blue and red dots in the PVP schematic denote nitrogen and oxygen heteroatoms, respectively.

In these 2D perovskites, large organic spacer cations form hydrogen bonds by intercalating between slabs of inorganic lead iodide octahedra, forming a layered perovskite structure (Figure 1). The “n” value represents the layers of lead iodide octahedra between cation layers. In this study, n = 1 perovskites are used for both their increased bandgap—therefore transparency—and improved stability [19]. For Ruddlesden–Popper (RP)-phase perovskites, the mono-ammonium group in the spacer cation (n-butylammonium, BA, in this study) participates in hydrogen bonding, with Van der Waals forces interacting between cation layers [20]. For Dion–Jacobson (DJ)-phase perovskites, a diammonium spacer cation (propane-1,3-diammonium, PDA, in this study) facilitates stronger hydrogen bonding between inorganic layers (Figure 1). Because of the bilateral hydrogen bonds present in diammonium-containing compositions, DJ perovskites are sometimes considered to be more structurally stable than RP [20]. However, published results reveal this is not absolute, and trade-offs exist in cation engineering for RP and DJ [17]. Nevertheless, 2D spacer cations demonstrate markedly improved operational and mechanical stability compared to 3D perovskites, particularly the n = 1 composition [19]. The key challenge for PV devices with 2D perovskites is to control the orientation of the layer such that the spacer cation does not hinder vertical charge transport. However, processing strategies such as hot casting have been demonstrated to preferentially control the orientation [21].

Additionally, additive engineering can be leveraged to further enhance the perovskite stability. The inclusion of the polymer polyvinylpyrrolidone (PVP) has been proven to induce compressive film stresses in CsPbI_3_ perovskite solar cells—a desirable effect associated with defect-healing properties [22]. Among mixed-halide perovskites such as CH_3_NH_3_PbI_3–x_Cl_x_, the presence of PVP is known to mitigate perovskite decomposition into PbI_2_ when the film is subjected to heat and moisture [23].

**Figure 2:**
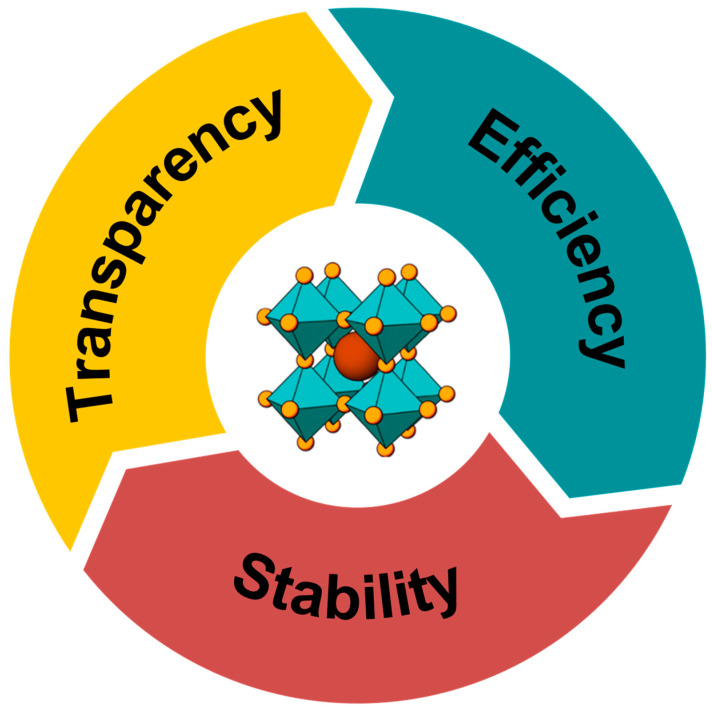
Transparency, Efficiency, and STability (TEST) Criteria. Suitability of BIPV absorber materials was gauged via these three characteristics.

To holistically evaluate the effects of compositional, dimensional, and additive engineering on the perovskite absorber material, we posit the use of three interrelated metrics: Transparency, Efficiency, and STability (TEST) (Figure 2). By examining these characteristics, we aim to understand which absorber composition and structure will best suit BIPV windows. The materials used in these devices must be transparent enough to let in ambient light to illuminate the inside of the building, which is quantified using UV–Vis spectroscopy. Additionally, to function as a light absorber, the material must also be able to generate electron-hole pairs from exposure to light. This is quantified using steady-state photoluminescence (PL). Lastly, to compete with conventional windows, a stable material that can withstand environmental stressors is imperative. Accelerated light and heat aging studies are performed to illustrate this with unencapsulated films. Ultimately, an effective absorber material for BIPV windows must perform well in all 3 of the TEST criteria to be considered a potential candidate. Of course, there remain additional interface and device engineering challenges that need to be considered, but for this study, film-level understanding remained the focus.

## 2. Materials and Methods

The RP inks were fabricated as follows: a total of 201 mg of n-butylammonium iodide (BAI) (Greatcell Solar, Queanbeyan, Australia, >99%) and 231 mg of lead iodide (PbI_2_) (TCI, Portland, OR, USA, >98.0%) were dissolved in 400 µL of N,N-dimethylformamide (DMF) (Sigma–Aldrich, St. Louis, MO, USA, 99.8%) and 600 µL of dimethyl sulfoxide (DMSO) (Sigma–Aldrich, ≥99.9%) to create a 0.5M solution of (BA)_2_PbI_4_ in 2:3 *v*/*v* DMF/DMSO. The 0.5M DJ samples were prepared in a similar fashion, dissolving 165 mg of propane-1,3-diammonium iodide (PDAI_2_) (Greatcell Solar, >99.8%) and 231 mg of PbI_2_ in 800 µL of DMF and 200 µL of n-methylpyrrolidone (NMP) (Sigma–Aldrich, 99.5%) to create PDAPbI_4_ in 4:1 *v*/*v* DMF/NMP. For the samples with polyvinylpyrrolidone (PVP), 4 wt.% of PVP (Sigma–Aldrich, 10,000 g/mol) was added with respect to the ink—61 mg for the RP–PVP ink and 57 mg for the DJ–PVP ink. Three-dimensional perovskites were fabricated as a basis to which the 2D samples were compared. For the 3D samples, a mixed-halide composition of 0.5M MAPbBr_1.5_I_1.5_ dissolved in 4:1 *v*/*v* DMF/DMSO was used. In this solution, 40 mg of methylammonium iodide (MAI) (Greatcell Solar, >99.99%), 28 mg of methylammonium bromide (MABr) (Greatcell Solar, >99.99%), 115 mg of PbI_2_, and 92 mg of lead bromide (PbBr_2_) (TCI, >98.0%) were dissolved in 800 µL of DMF and 200 µL of DMSO.

All films in this study were solution-processed and deposited onto 1″ × 1″ glass substrates via spin coating in an N_2_ glovebox. Prior to spinning, all substrates were preheated at 100 °C for 10 min. The films were spin-coated at 4000 rpm for 30 s, with chlorobenzene (CB) (Sigma–Aldrich, 99.8%) antisolvent dropped at 15 s for only the 3D films. After spinning, all films were annealed at 100 °C for 10 min on a hot plate. The produced films were then subject to accelerated light and heat aging under AM 1.5G illumination and 85 °C, respectively, for 96 h.

Microscope images were taken on a Keyence VHX-7000 (Itasca, IL, USA). A Cary 5000 UV–Vis-NIR spectrophotometer (Santa Clara, CA, USA) was used to collect absorption and transmission spectra. For the steady-state PL data, the CPS450 collimated 450 nm, 4.5 mW laser diode module was used. The Rigaku Smart Lab X-ray Diffractometer (Tokyo, Japan) was used to gather XRD spectra.

## 3. Results

**Figure 3:**
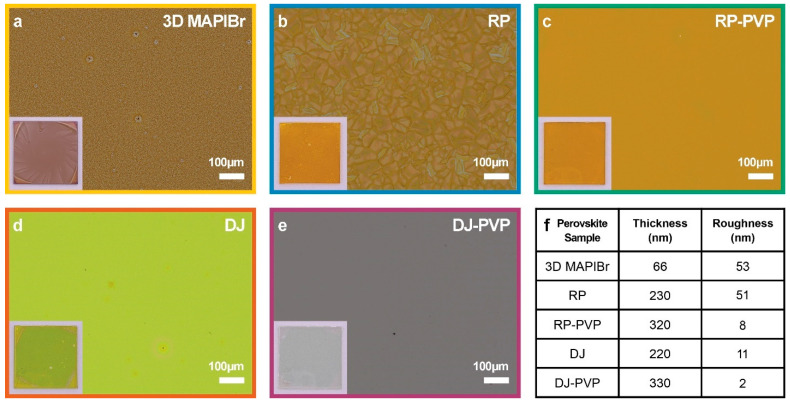
Morphology, Thickness, and Roughness. (**a**–**e**) Optical microscope images of 3D, RP, RP–PVP, DJ, and DJ–PVP films, respectively. Inset are camera images of the 1″ × 1″ samples. (**f**) Table of thickness and roughness values for each sample.

A one-step spin coating process without quenching was used to produce uniform films of both n = 1 DJ (PDAPbI_4_) and n = 1 RP ((BA)_2_PbI_4_) perovskites featuring good surface coverage and no discernable pinholes. Despite employing the commonly used antisolvent method [24] for the 3D mixed-halide samples (MAPbBr_1.5_I_1.5_), localized islands of nonuniformity remained (Figure 3a). These features are likely caused by nonuniform crystallization and were not present in either the DJ or RP films, a challenge that has been well-documented for mixed-halide 3D perovskite films [25]. For the RP film in Figure 3b, features tens of microns across can be observed, which are not visually evident in the RP–PVP films (Figure 3c). Guo et al. suggest that this may be due to the PVP causing the perovskite to form nanocrystals, which result in smaller surface features [23]. These features are small enough such that they are indistinguishable at millimeter length scales. Consequently, this resulted in a reduction in the roughness of the RP–PVP and DJ–PVP films, with the DJ–PVP films reaching an extremely low roughness of 2 nm (Figure S1). In addition to reducing the roughness, PVP increased the perovskite film thickness (Figure S2). Adding PVP resulted in films roughly 100 nm thicker than without it for both DJ–PVP and RP–PVP samples. Thickness and roughness values are summarized in Figure 3f.

While the 3D films were the characteristic brown color of mixed-halide perovskites [13], the RP films exhibited a mild orange color—regardless of whether PVP was added (Figure 3b,c inset). However, for the DJ films, a drastic color change from neon green (Figure 3d) to nearly transparent (Figure 3e) can be seen when incorporating the additive—an effect that is attributed to the increased bandgap of the DJ–PVP films [12]. This shows the potential for using 2D perovskites in designing BIPV for aesthetics with compositional tuning to adjust the color.

**Figure 4:**
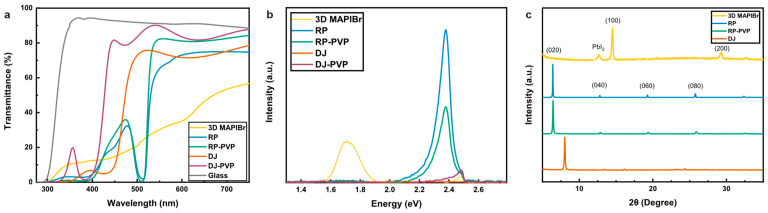
Optoelectronic and Structural Properties. (**a**) UV–Vis transmittance spectra of all five samples, including glass, were collected with air as the baseline. (**b**) Steady-state PL curves. (**c**) XRD spectra of 3D, RP, RP–PVP, and DJ samples. The DJ–PVP spectrum was not included due to issues in crystallization.

The color and transparency changes observed in the films were measured with UV–Vis spectroscopy (Figure 4a). In the absorption and transmission spectra of the DJ and RP films, oscillations can be observed, which may be attributed to interference effects (Figure S3). Additionally, they displayed a much higher transmittance than their 3D counterpart, which had the poorest AVT of 31%. In contrast, the DJ film had a reasonable AVT of 55%, which increased to 72% AVT with the DJ–PVP. The increased transparency was met with an expected blue-shift in the bandgap from 2.63 eV to 2.95 eV, as derived from Tauc plots of the absorbance data. The RP films reached an AVT of 47%, which increased to 54% AVT with the RP–PVP films. These RP and RP–PVP samples exhibited similar absorption profiles for the sub-523 nm wavelengths (Figure 4a). For longer wavelengths, however, RP–PVP samples had improved transmission, resulting in nearly a 10% increase in AVT compared to the RP. Despite the difference in AVT, there was no significant change in the bandgap energy between the two types of RP films, with RP and RP–PVP films having a measured bandgap of 2.37 eV and 2.38 eV, respectively. As such, the PVP appears to have a more prominent role in affecting the DJ structure than the RP perovskite.

The relatively lower measured PL intensity of the 3D perovskite seen in Figure 4b is likely due to a combination of reduced thickness, poorer morphology, incomplete film coverage of the substrate, and the well-documented temporal instability of the PL intensity in mixed-halide perovskites [26]. This peak instability was not observed for the pure-iodide RP samples, which is an indication of the improved optoelectronic stability of the RP perovskite. Among the RP samples, the RP–PVP exhibited a lower PL response than the RP film. Because PL intensity can be used as a proxy for electrical performance [26], we conclude that PVP slightly reduces optoelectronic response due to the insulating nature of the material. However, previous work on all-inorganic perovskite suggests that PVP-enhanced perovskite films can reap the benefits of the additive without compromising device performance [27], and tuning the concentration of the PVP is a direction that will be studied in future work.

In stark contrast, neither of the DJ samples yielded any noticeable PL response when excited with the same laser diode. Despite the remarkable transparency displayed by the DJ–PVP films, the low PL signal suggests poor light-harvesting performance in a solar device. Since a 450 nm (~2.76 eV) laser was used for excitation, it is possible that a shorter excitation wavelength would be needed to achieve a better response, particularly for the DJ–PVP sample. Nonetheless, it is important to note that at these extremely wide bandgaps, the theoretical power conversion efficiency limit is quite low (~5%).

X-ray diffraction (XRD) spectra for the RP perovskites in Figure 4c show a dominant reflection of the (020) plane, with additional peaks at (040), (060), and (080), which aligns with previously reported data from Lin et al. [28]. On the subject of phase stability with the RP–PVP films, no new peaks appear—only a slight broadening likely caused by the polymer’s amorphous structure. Overall, the RP phase remains intact despite the presence of PVP, an effect which shows the PVP does not disrupt the overall 2D perovskite crystallinity. This is reinforced by the minimal change in bandgap between the RP and RP-PVP films.

Similar to the RP films, there is also a prominent diffraction peak at low angles within the 5°–10° range for the DJ, aligning well with the structure of 2D perovskites. However, the XRD data reveals that the addition of PVP to the DJ–PVP ink resulted in films that did not crystallize to the perovskite phase. This may be studied further via concentration studies to identify an ideal concentration of PVP that maintains the desired phase; however, this was not a focus of this study since the unmodified DJ film did not show a noticeable PL response.

For the 3D spectra, there is a small PbI_2_ peak observed at 12.7°, characteristic of perovskite degradation as well as poorer crystallization in the thin film (Figure 4c). This is further supported by the increased peak widths in the 3D sample when compared to the spectra from the DJ and RP films.

To better understand the environmental stability characteristics of the perovskite films, they were subjected to accelerated light and heat aging. PL spectra were measured periodically in 24-h intervals over 96 h. Because of the minimal performance of the DJ films under PL, they were not used in this aging study. As such, the following discussion on film stability will focus on the comparison between the performance of the RP and 3D films.

**Figure 5:**
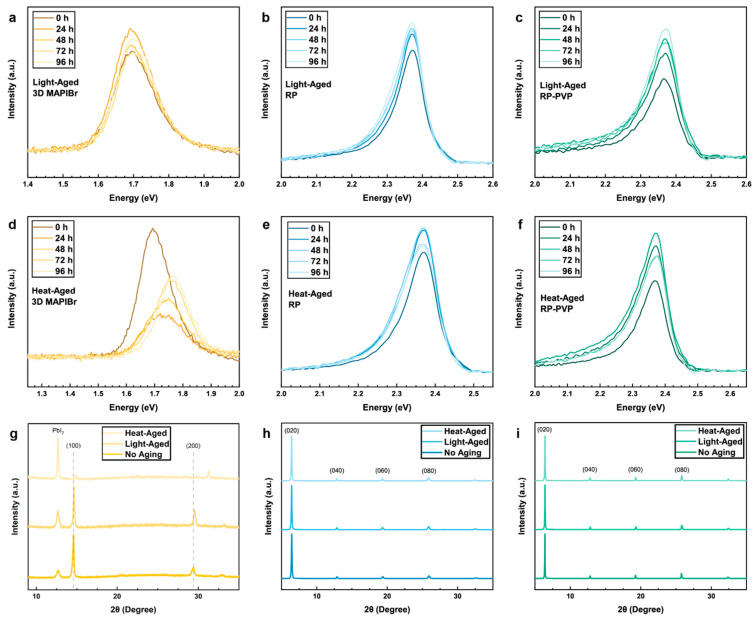
Light and Heat Aging Studies. (**a**–**c**) Steady-state PL spectra of 3D, RP, and RP–PVP films subject to 1 sun illumination for 96 h, collected in 24-h intervals. (**d**–**f**) Steady-state PL spectra of films subject to 85 °C accelerated thermal aging for 96 h, collected in 24-h intervals. (**g**–**i**) XRD spectra of films before and after the aging studies.

The RP perovskite demonstrated superior stability over the 3D film under illumination for both RP and RP–PVP samples (Figure 5a–c). The maximum intensity of the 3D PL peak begins to decrease after only 48 h, whereas the RP continues to steadily increase over the 96-h aging period. Additionally, the PVP serves to bolster this stability, causing a 66.8% increase in PL intensity after aging (Figure 5c, Table 1). The larger increase in PL may be attributable to a reduction or annihilation of trap states [29,30].

Under thermal aging, it is evident that all samples suffered from thermal exposure to high heat (Figure 5d–f). The PL intensity of the 3D film diminished by 63.7% (Figure 5d, Table 1). This is expected, as it is well-documented that 3D perovskites are susceptible to degradation under heat [31], particularly methylammonium-based compositions used here. Further evidence of phase segregation is observed in the blue-shift of the PL peak from 1.72 eV to 1.74 eV. As the Pb-I bonds break with increased thermal stress, the Pb-Br bonds remain, resulting in PL characteristics more aligned with that of a pure methylammonium lead bromide perovskite, thus the shift to higher energies [30].

A drastic blue-shift was not seen for either RP or RP–PVP heat-aged films, where the PL emission remained consistent at roughly 2.37 eV. Unlike the light-aged films, whose PL intensities increased steadily, the PL intensity of the heat-aged RP films began to drop after 72 h. This indicates that the RP perovskite used in this study is more resilient to light than thermal stressors. While PVP does not solve this instability, it does, however, result in a 29% increase in intensity overall (Figure 5e, Table 1). In comparison, the PL increased only 5% in the absence of PVP (Figure 5f, Table 1). The increase in PL intensity suggests enhanced thermal stability due to the addition of PVP.XRD spectra were collected after the aging study to complement the PL data and to analyze structural changes that may have occurred (Figure 5g–i). The 3D samples experienced the most degradation due to heat. These films clearly exhibit degradation of the perovskite into lead iodide, as supported by the growth of the peak at 12.7°, particularly for the heat-aged sample. Further evidence of degradation is seen in the loss and shift of the perovskite peak originally at 14.6° in the heat- and light-aged samples, respectively (Figure S4). There is also possible evidence for a stress relaxation-based degradation mode in the heat-aged sample, in particular, based on the larger shift in 2θ for the (200) compared to the (100) peak.

There is no noticeable growth of the lead iodide peak at 12.7° with the RP samples, nor is there an indication of stress relaxation under either light or heat. This suggests that the RP perovskite retains its structure even after being subjected to accelerated aging. Similar results were observed in the RP–PVP, whose spectra indicate strong phase stability after aging. While there is a slight peak shift to lower 2θ, the change is negligible and on the order of hundredths of a degree (Figures S5 and S6). For the RP–PVP films, in particular, the light-aged samples underwent a larger shift than those aged with heat (Table S1).

Overall, it is evident from the PL and XRD spectra that the RP thin films exhibit superior light and thermal stability when compared to traditional 3D perovskites. Their phase stability and resistance to degradation are ideal for their use in BIPV.

**Figure 6:**
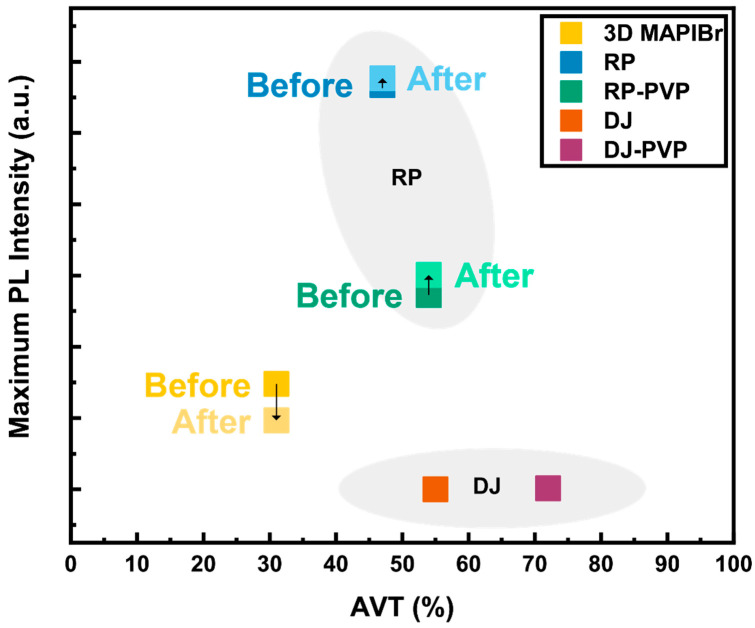
The Metric. A visual representation of the candidate materials’ suitability for BIPV windows based on the TEST criteria. Maximum PL intensities vs. AVT for each sample are plotted both before and after aging with heat (85 °C) and demarcated accordingly.

To summarize our findings, we developed a metric that visually represents the suitability of the materials we studied for BIPV windows based on the TEST criteria (Figure 6). The RP films yielded the best balance among the three—achieving respectable transparency, great PL, and strong phase stability under light and heat. On the other hand, the DJ and 3D films were deemed less suitable for their poor PL and stability characteristics, respectively.

## 4. Discussion

In this work, we leveraged the TEST criteria to evaluate the suitability of DJ and RP perovskite materials for their use in BIPV windows. Ultimately, we propose the use of RP phase 2D perovskites with a PVP additive as a promising candidate for the absorber material in BIPV applications. While the DJ films held a higher AVT, the trade-off lies in their weak PL characteristics. In contrast, RP–PVP films exhibited a respectable AVT (>50%) with strong PL and structural stability, as quantified by PL and XRD measurements under accelerated aging studies. In conclusion, our study highlights the potential of RP perovskites as a stable, semitransparent absorber material for BIPV. We have successfully demonstrated the viability of these perovskite compositions, although further work is needed to integrate these films into semitransparent devices effectively.

Moving forward, future experiments will focus on studying charge transport layers, electrodes, and their interfacial interactions within semitransparent perovskite PV devices. Additionally, understanding the interaction of PVP with perovskite films through concentration studies is crucial for optimizing device performance.

This study ultimately adds to the growing body of work revolving around 2D/low-dimensional perovskites. This not only expands the scope of semitransparent solar cells but also opens avenues for other applications in optoelectronics. More stable and transparent perovskites can unlock the potential of existing building facades and open new frontiers in the field of solar energy. Overall, our work lays the groundwork for future innovations in the field, driving us closer to ubiquitous and lasting energy solutions.

## Figures and Tables

**Table 1:** Change in pre- and post-aging PL intensity for films aged under light and heat over a 96-h period.

Aging	3D MAPIBr	RP	RP–PVP
Light-aged	14% increase	25% increase	66% increase
Heat-aged	64% decrease	5% increase	29% increase

## Data Availability

Data will be made available upon reasonable request.

## Supplementary Materials

The following supporting information can be downloaded at: https://www.mdpi.com/article/10.3390/nano14141184/s1, Figure S1: Roughness of perovskite thin films measured via profilometry (a) 3D, (b) RP, (c) RP-PVP, (d) DJ, (e) DJ-PVP films; Figure S2: Thickness of perovskite thin films measured via profilometry for (a) 3D, (b) RP, (c) RP-PVP, (d) DJ, (e) DJ-PVP films; Figure S3: Absorbance profile of perovskite thin films including glass. Absorbance is measured with respect to air as the baseline; Figure S4: Zoomed in X-ray diffraction spectra from Figure 5 for the (a) (001) PbI2 and (100), (b) (200) peaks, showing evidence for stress relaxation in the thermally aged 3D perovskite samples; Figure S5: Zoomed in X-ray diffraction spectra from Figure 5 isolating each peak in the RP samples corresponding to the (a) (020), (b) (040), (c) (060), (d) (080) peaks. The data showcases phase stability after accelerated aging. Peaks shift slightly (<0.1°) to lower angles; Figure S6: Zoomed in X-ray diffraction spectra from Figure 5 corresponding to the (a) (020), (b) (040), (c) (060), (d) (080) peaks. The data shows that the RP phase is maintained even when adding PVP. Further, the RP-PVP samples largely retain phase stability even after accelerated aging. Peaks shift slightly (<0.1°) to higher angles; Table S1: XRD peak shifts after light and heat exposure, showing evidence for stress relaxation in the heat-aged 3D film and minor change in the RP and RP-PVP films.
