# Supplementary material for: 2D Ruddlesden–Popper Perovskites with Polymer Additive as Stable and Transparent Optoelectronic Materials for Building-Integrated Applications
Source: Nanomaterials (Basel). 2024 Jul 11;14(14):1184. doi: 10.3390/nano14141184 (PMC11280112; doi:10.3390/nano14141184)
Supplement: Supplementary file 1 [file nanomaterials-14-01184-s001.zip › nanomaterials-3060642-supplementary.pdf]

Supplementary Materials

2D Ruddlesden–Popper Perovskites with Polymer Additive as Stable and Transparent Optoelectronic Materials for Building-Integrated Applications

Adianne Alamban, Muneeza Ahmad and Nicholas Rolston *

Renewable Energy Materials and Devices Lab, School of Electrical, Computer and Energy Engineering (ECEE), Arizona State University, Tempe, AZ 85284, USA; aalamban@asu.edu (A.A.); mahmad15@asu.edu (M.A.)
* Correspondence: nicholas.rolston@asu.edu

Keywords: perovskite solar cells; defects; 2-dimensional perovskite; structure; strain; semitransparent; wide bandgap

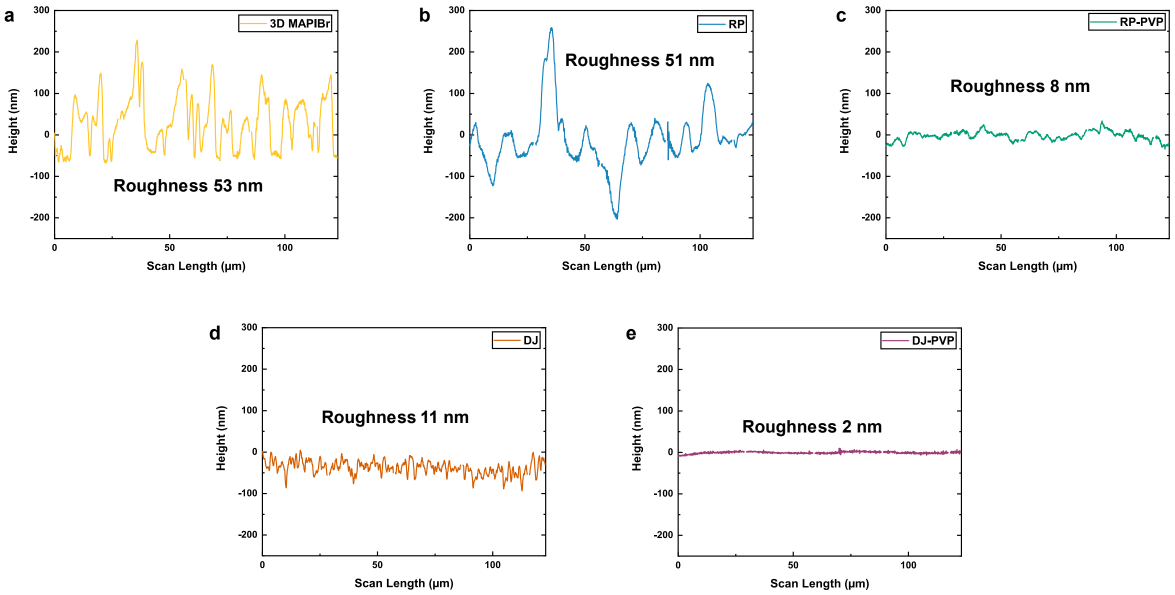

Figure S1. Roughness of perovskite thin films measured via profilometry **a** 3D, **b** RP, **c** RP-PVP, **d** DJ, **e** DJ-PVP films.

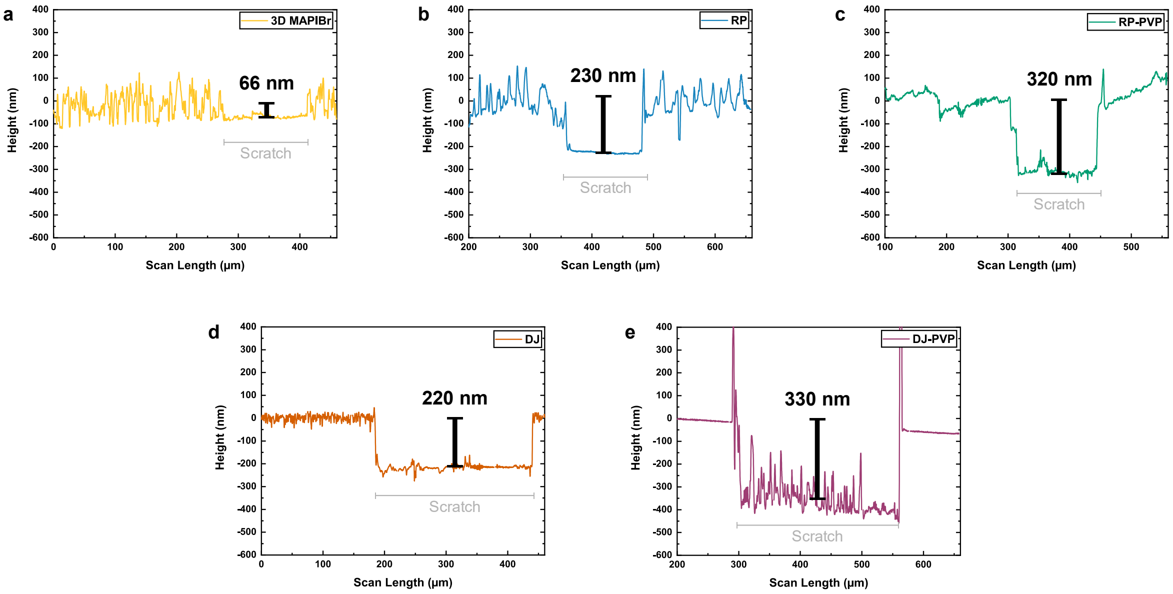

Figure S2. Thickness of perovskite thin films measured via profilometry for **a** 3D, **b** RP, **c** RP-PVP, **d** DJ, **e** DJ-PVP films.

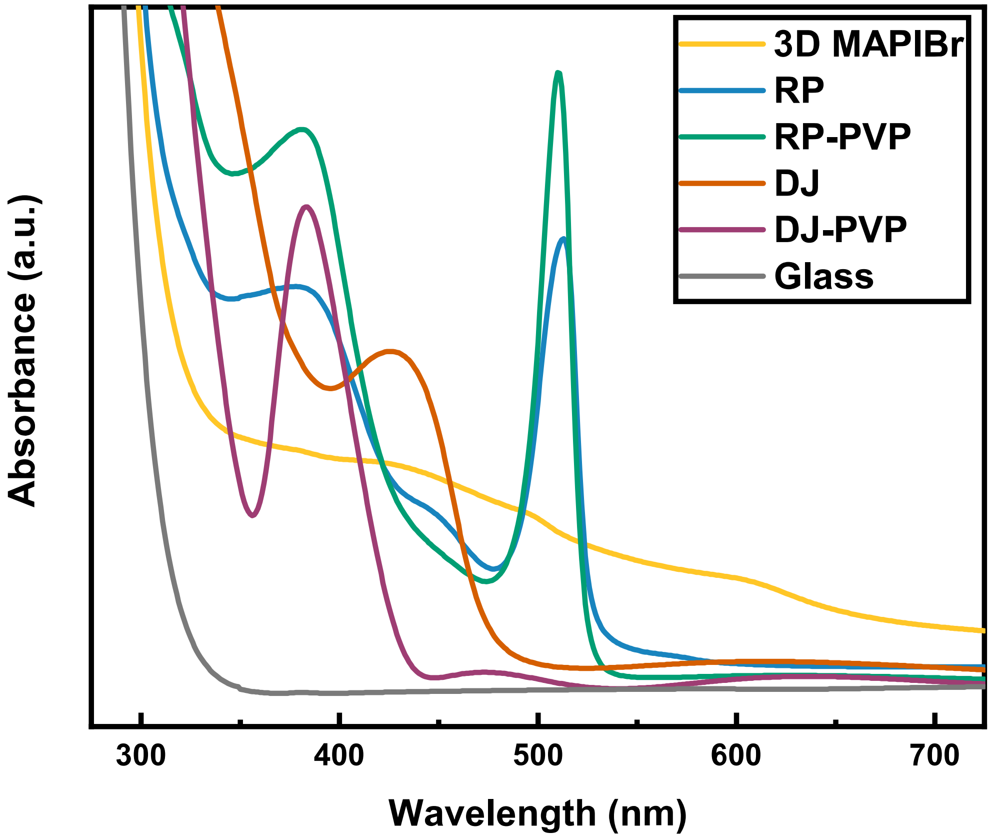

Figure S3. Absorbance profile of perovskite thin films including glass. Absorbance is measured with respect to air as the baseline.

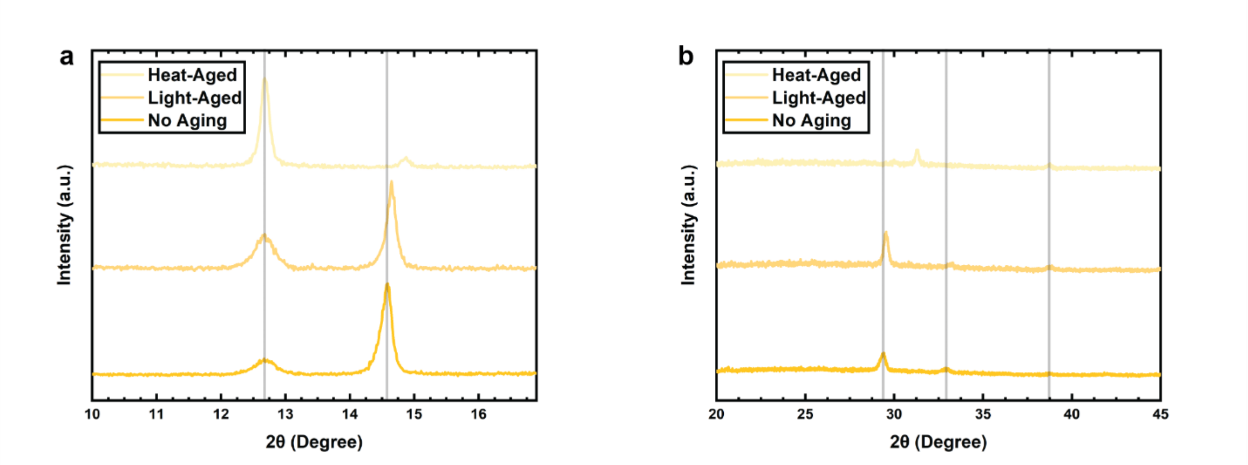

Figure S4. Zoomed in X-ray diffraction spectra from **Figure 5** for the **a** (001) PbI_2 and (100), **b** (200) peaks, showing evidence for stress relaxation in the thermally aged 3D perovskite samples.

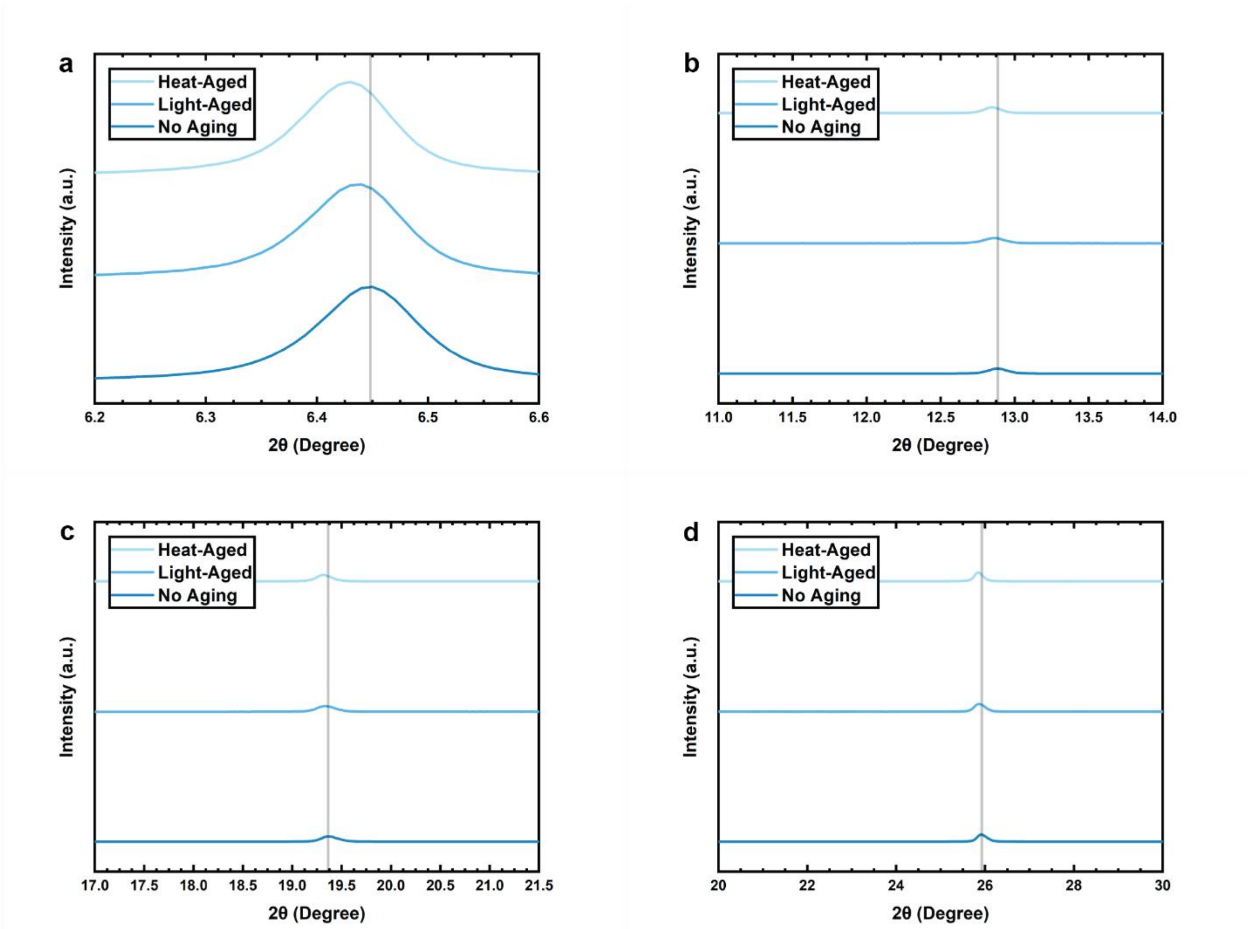

Figure S5. Zoomed in X-ray diffraction spectra from **Figure 5** isolating each peak in the RP samples corresponding to the **a** (020), **b** (040), **c** (060), **d** (080) peaks. The data showcases phase stability after accelerated aging. Peaks shift slightly (<0.1°) to lower angles.

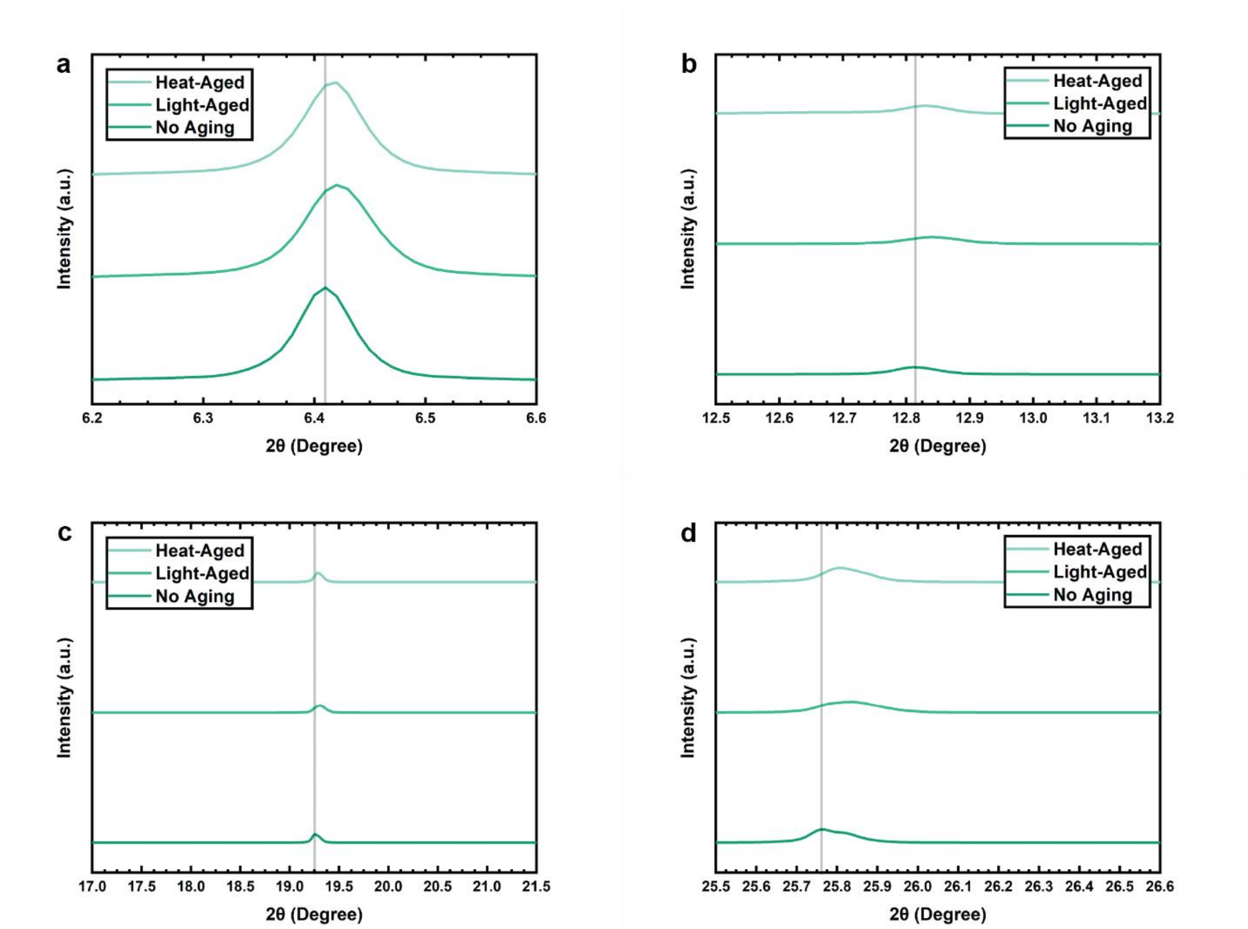

Figure S6. Zoomed in X-ray diffraction spectra from **Figure 5** corresponding to the **a** (020), **b** (040), **c** (060), **d** (080) peaks. The data shows that the RP phase is maintained even when adding PVP. Further, the RP-PVP samples largely retain phase stability even after accelerated aging. Peaks shift slightly (<0.1°) to higher angles.

3D XRD Peak Shift

Peak	Light	Heat
(001) PbI_2	0°	0°
(100)	6.84 E-3°	20.5 E-3°
(200)	6.80 E-3°	61.2 E-3°

RP XRD Peak Shift

Peak	Light	Heat
(020)	-1.55 E-3°	-3.10 E-3°
(040)	-2.32 E-3°	-3.10 E-3°
(060)	-2.57 E-3°	-3.61 E-3°
(080)	-2.31 E-3°	-3.08 E-3°

RP-PVP XRD Peak Shift

Peak	Light	Heat
(020)	1.56 E-3°	1.56 E-3°
(040)	2.34 E-3°	2.34 E-3°
(060)	2.59 E-3°	1.55 E-3°
(080)	2.71 E-3°	1.94 E-3°

Table S1. XRD peak shifts after light and heat exposure, showing evidence for stress relaxation in the heat-aged 3D film and minor change in the RP and RP-PVP films.
